# Words describing feelings about death: A comparison of sentiment for self and others and changes over time

**DOI:** 10.1371/journal.pone.0242848

**Published:** 2021-01-06

**Authors:** Lauren R. Miller-Lewis, Trent W. Lewis, Jennifer Tieman, Deb Rawlings, Deborah Parker, Christine R. Sanderson

**Affiliations:** 1 Research Centre for Palliative Care, Death and Dying, Palliative and Supportive Services, College of Nursing and Health Sciences, Flinders University, Adelaide, South Australia, Australia; 2 Department of Psychology and Public Health, School of Health, Medical and Applied Sciences, CQUniversity Australia, Adelaide Campus, Wayville, South Australia, Australia; 3 College of Science and Engineering, Flinders University, Adelaide, South Australia, Australia; 4 Faculty of Health, University of Technology Sydney, Ultimo, New South Wales, Australia; 5 Palliative Medicine, Calvary Health Care Kogarah, Kogarah, New South Wales, Australia; 6 Territory Palliative Care–Central Australia, Alice Springs Hospital, The Gap, Northern Territory, Australia; University of Vermont, UNITED STATES

## Abstract

Understanding public attitudes towards death is needed to inform health policies to foster community death awareness and preparedness. Linguistic sentiment analysis of how people describe their feelings about death can add to knowledge gained from traditional self-reports. This study provided the first description of emotive attitudes expressed towards death utilising textual sentiment analysis for the dimensions of valence, arousal and dominance. A linguistic lexicon of sentiment norms was applied to activities conducted in an online course for the general-public designed to generate discussion about death. We analysed the sentiment of words people chose to describe feelings about death, for themselves, for perceptions of the feelings of ‘others’, and for longitudinal changes over the time-period of exposure to a course about death (*n* = 1491). The results demonstrated that sadness pervades affective responses to death, and that inevitability, peace, and fear were also frequent reactions. However, words chosen to represent perceptions of others’ feelings towards death suggested that participants perceived others as feeling more negative about death than they do themselves. Analysis of valence, arousal and dominance dimensions of sentiment pre-to-post course participation demonstrated that participants chose significantly happier (more positive) valence words, less arousing (calmer) words, and more dominant (in-control) words to express their feelings about death by the course end. This suggests that the course may have been helpful in participants becoming more emotionally accepting in their feelings and attitude towards death. Furthermore, the change over time appeared greater for younger participants, who showed more increase in the dominance (power/control) and pleasantness (valence) in words chosen at course completion. Sentiment analysis of words to describe death usefully extended our understanding of community death attitudes and emotions. Future application of sentiment analysis to other related areas of health policy interest such as attitudes towards Advance Care Planning and palliative care may prove fruitful.

## Introduction

The outsourcing of dying and death from families to professional settings in contemporary western societies appears to have made reluctance to think, talk, or communicate about death normative [1, 2]. This is particularly problematic given the anticipated future social, economic, and personal challenges resulting from the ageing population and its corresponding rise in death rates. Thus, planning for how we live and provide care at the end-of-life is becoming a major public health issue [3, 4]. Understanding community attitudes is necessary for engaging in dialogue about dying and death, and for how we manage future needs and expectations of care at the end-of-life [3]. However, the current evidence base is limited and there have been calls for more rigorous research to better capture public views on dying and death. Understanding contemporary community attitudes and feelings towards death is valuable because it may inform the development of future health services, improve patient and family care at the end-of-life, and it may also inform policy on ways to encourage conversations leading to greater death preparedness and planning [3, 5–7].

The language we use plays a central role in these conversations about death and dying. Words matter because ones’ attitude towards a topic is often implicit in lexical choice–words used are reflective and expressive of our personal attitudes and emotions, and can also potentially influence how we think and lead to attitudinal change [8–11]. The words people use reflect not only what they are thinking about, but also how they are feeling, which offers insight into their emotional lives, and therefore analysis of words can be a useful addition or complement to traditional self-reports of emotion [12–16]. Thus a novel way to examine the attitudes of people towards an issue is to analyze the types of words they use to describe it, and the linguistic sentiment inherent in those words [11]. Indeed, multidisciplinary research has demonstrated through text analysis that word use is a reliable indicator of a word-users’ psychological state [17].

More specifically, ‘sentiment analysis’ analyses people’s evaluations, appraisals, attitudes, and emotions towards entities such as products, services, issues and topics [18]. Most sentiment analysis work focuses on detecting the affect or emotion of text and focusses on assigning a positive or negative rating (valence) to that text [19].

Despite the potential arising from computerized advancements in text and sentiment analysis technology that enable reliable psycholinguistic assessment of the language people use, word use and sentiment remains a relatively understudied phenomenon [14] in relation to health issues. Until recently, word sentiment has been neglected in the context of death and dying, even though there have been calls for the use of alternative methods to investigate attitudes towards dying [7]. The recent linguistic works of Gramling and Gramling [20] on clinical cancer palliative care conversations [21], and Semino and colleagues [22] on metaphors used in cancer and end-of-life discussions are notable exceptions. For example, health professionals’ metaphors for ‘good’ and ‘bad’ deaths were examined, and detected contrasting metaphors of ‘peace’, ‘freedom’, ‘openness’ and ‘acceptance’, compared to ‘struggle’ and ‘pushing away’ [22, 23]. Also, the sentiment of words in palliative care consultations moved from sadder to happier valences through the course of the conversation arcs between dyads of cancer patients and clinicians [21].

Some other smaller studies have examined word use in relation to the topic of death. Qualitative studies have demonstrated the valuable role of words in narratives for understanding attitudes related to meaning-making in death and palliative care [24–26]. One qualitative study of the emotions aroused by the thought of one’s own death in young nursing students found that fear, pain, anguish, and sadness were the most commonly reported emotions [27]. A series of quantitative studies about death have utilized elements of the Linguistic Inquiry and Word Count (LWIC) database [28]. Evans, Walters and Hatch-Woodruff [29] asked undergraduates to write a narrative about their own hypothetical death or the death of another, and found writing about the death of another was associated with more realistic considerations of death (pain, negative emotions). A related series of experiments by Kashdan and colleagues [30] found that undergraduates asked to write a narrative about their own hypothetical death used more positive emotion words compared to those who wrote about dental pain, perhaps to direct attention away from the threat of their mortality. Concurring with this, linguistic analysis of blog posts by people with terminal illness showed less use of negative affect words and more positive affect words than seen in simulated blogs by non-patients imagining imminent death [16]. Another study [12] used linguistic analysis of words used in bereavement meetings between physicians and bereaved parents, and found that parents used more positive emotion words and less negative emotion words over the course of the meeting, indicating the benefit of the meeting for parents. Positive and negative affect linguistic predictors of adaptive bereavement and other mental health conditions have also been examined [31, 32], as has the sentiment expressed over time in text written in the lead-up to suicide, showing an increase in positive emotions expressed [33].

One restriction within these previous studies is the singular focus on the level of positive and negative affective sentiment of words. Osgood, Suci, and Tannenbaum’s Theory of Emotions (and applications thereof) state that there are three dimensions that together sufficiently and adequately describe the spectrum of people’s emotional responses to stimuli of all types [19, 34–36]. The first, and most important emotional dimension is ‘Valence’, which covers the affective response to a stimulus on a continuum from unhappy/unpleasant to happy/pleasant. The second dimension is the ‘Arousal’ evoked by a stimulus, based on the spectrum of activation or alertness, ranging from feeling calm to excited. The third dimension is ‘Dominance’ or power of the stimulus, which represents the continuum of control, and ranges from feelings of being controlled externally (submissive, lacking influence) to a feeling of being in control (dominating and feeling influential), and has been likened to the concept of locus of control [34–36]. The application of these three emotional dimensions to stimuli related to language and communication, and specifically to words used, provides a rich source of insight into the emotional state of people [19, 34, 35, 37].

Large-scale sentiment analysis of words has been conducted to develop norms that can distinguish between these three components of emotions in line with the Theory of Emotions [37]. A computational text-based database developed by Warriner and colleagues [37] provides norms on valence, arousal, and dominance ratings for the majority of well-known English language words (13,915 words), building upon the initial norms collected by Bradley and Lang’s [38] Affective Norms for English Words (ANEW) database for 1034 words. Warriner’s and colleagues database [37] is considered representative of well-known root words in English, and covers a tremendous number of emotional words, thus providing a very rich source of information on the affective meaning of words, and the emotional connotations inherent in their use [39]. Indeed, its stated that Warriner and colleague’s corpus “provides a unique contribution to advance the field of emotion research” [39] (p.150). Application of these ratings to the language used by people can therefore provide a more objective insight into the inner emotional state of the word-user.

The valence and arousal model, often called the “circumplex model” of affect [40] is widely used in psychological and psycholinguistic studies [41]. In computational linguistics, this model is applied when the interest is in continuous measurements of valence and arousal rather than in the specific discrete emotional categories [42]. A frequently used tool in the area of computational linguistics for studying text is LIWC which provides an analysis of writing style, including psychologically-grounded lists of positive and negative emotional terms [13]. However, this tool is devised for passages of text and only labels text as positive or negative discrete emotion. Warriner’s lexicon [37] offers the advantage of continuous measurement ratings on all three key dimensions of emotion for an extensive volume of English words.

The valence, arousal, and dominance sentiment of words used to express feelings related to death is a largely neglected area of research. A study using the ANEW lexicon indicated that words about death have lower valence and higher arousal than words in more general categories [43]. Images of death have also been assessed using ANEW-related Self-Assessment Manikin ratings, and found similar effects [44, 45]. Interestingly, images of death elicited less arousal in people who had prior experience with death during healthcare training [45]. To our knowledge, no studies have applied Warriner and colleagues [37] lexicon norms for valence, arousal and dominance specifically to the topic of death.

Nonetheless, researchers have used this type of sentiment analysis to successfully examine other clinical phenomena. Studies have applied Warriner and colleagues sentiment lexicon, or its precursor ANEW, to accurately detect the presence of depression and other mental disorders from text and speech transcripts [46–51]. For example, clinically-affected groups tended to use words with lower valence in their online posts [47]. Studies have also examined the valence and arousal of words that convey pain [52], and the valence, arousal and dominance of emotional reactions to therapeutic associative COPE cards used in counselling [53]. Thus, there is potential value in conducting sentiment analysis of valence, arousal and dominance within the context of death and dying.

### Research objectives

The application of sentiment analysis to death and dying attitudes and emotions represents a unique opportunity to form a deeper understanding about how the public perceive this topic, which is increasing importance to health policy and service delivery. Textual sentiment analysis of how a person describes death can build upon knowledge gained from more traditional self-report measures like surveys [5–7, 54–56] and enable a more comprehensive triangulation of this phenomenon [29].

Within an online learning environment, language plays an important role given it is the vessel through which learners virtually interact and exchange their thoughts and feelings [9, 57]. Particularly for emotion-laden topics like death, linguistic sentiment analysis of the words online course participants choose can provide rich insights into the underlying meaning and attitudinal connotations of their language, and how this may change over the course of an online learning experience. With this in mind, we applied linguistic sentiment methodology to activities conducted in a Massive Open Online Course (MOOC) designed for the general public to generate discussion about death and dying, with the intention of facilitating community acceptance of death as a natural part of life. Specifically, activities in the introductory week of the MOOC asked participants to choose three words that best describe how they personally feel about death and dying. Next, they were asked to choose three words that they think best describe how *others* in the general public feel about death and dying. Then five-weeks later as a final activity at the conclusion of the MOOC, participants were asked again to choose three words that best describe their own feelings about death and dying. Given that it is estimated that less than five percent of the words people use in daily speech and writing can be classified as emotional [14], these activities were designed in a way to encourage the considered choice of emotional words in relation to death. Using text analysis of multiple written comments may have been hampered by this typical lack of emotional word use, making it potentially difficult to detect differences over time or between self-other perceptions.

Sentiment analysis of the responses to these MOOC activities can potentially provide useful insights into personal attitudes towards death and how these might alter through participation in a course focused on the topic. It can additionally inform our understanding of individuals’ perceptions of how other people feel about death, for which there is little knowledge in the literature. Our previous research has found the majority of participants enrolling in a course about death agreed with the statement that most people do not comfortable talking about death [5], and that the use of euphemisms was common [58]. A study with elderly parent and middle-age child dyads found that the children significantly overestimated their parent’s level of death anxiety, rating it as higher than their own when in fact it was lower [59]. A qualitative study focusing on the oldest-old found similarly accepting attitudes towards death and its discussion, but that in several cases the younger family member’s perceptions of their elderly relatives’ attitudes towards death contrasted with the actual attitudes and preferences expressed by them [60]. Thus, understanding self-other perceptions may be useful implications for encouraging end-of-life conversations.

The present study had two key research objectives. First, we aimed to describe the types and sentiment of words that MOOC participants used to describe their personal feelings about death, and compare them to the words they attributed to the feelings of other people in the general community (e.g., was there differences in the way participants’ described death from their own perspective versus the perspective of others?) Second, we aimed to determine if there was a change over time in the types and sentiment of words participants chose to describe how they feel about death from the beginning of the MOOC to the end of the MOOC. It was expected that MOOC participants’ chosen words to describe their own feelings about death would have a more positive sentiment (increased valence and dominance and reduced arousal) at the end of the MOOC course than they did at the beginning. We also aimed to explore whether participant’s socio-demographic characteristics were associated with the sentiment of the words chosen to describe death, or associated with the level of change over time in word sentiment.

## Materials and methods

### Study design and setting

The methods utilised to conduct this study are described in accordance with the Strengthening the Reporting of Observational Studies in Epidemiology (STROBE) statement [61]. This study was conducted as part of the Dying2Learn MOOC about death and dying, which was delivered on the OpenLearning online education platform.

The design of the present study included descriptive content frequency analysis of words used, and the application of lexical analysis tools to assign numeric sentiment values to words. These were then subsequently used in quantitative analyses to determine if significant group differences (cross-sectional) and change over time (longitudinal) existed.

### Participants

The Dying2Learn MOOC was freely available to the general public on the OpenLearning platform, with no eligibility restrictions placed on enrolment. This study is based on data from both the 2016 and 2017 cohorts of the MOOC. A total of 1,156 people enrolled in the Dying2Learn MOOC in 2016, and 1,960 enrolled in 2017. Of the 3116 people who completed the MOOC enrolment form, a total of 35.9% (n = 1118) never entered or commenced participation in the MOOC. A total of 13.8% completed at least 90% of all the activities in the MOOC. This ‘funnel of participation’ is a typical characteristic of MOOCs, where after enrolment there is a steep decline in active engagement (with less than half of those who enrol commencing any participation at all), and only a small proportion MOOC enrolees complete the MOOC (around 12–15%) [62–64].

The present study is based on data from the subset of *n* = 1491 MOOC participants who engaged with activities asking them to choose three words to describe death. A total of 1350 participants completed both the three-words activities in the introductory week of the MOOC, which involved reporting (a) words to describe both how they personally feel about death *and* (b) words they think describe how the general public might feel (i.e., words that conveyed their perceptions about other people’s feelings towards death). This represented 43.33% of all people who enrolled in the MOOC over the 2 years. At the conclusion of the MOOC in the final reflections week, participants were asked to revisit the three-words activity, and again choose three words to describe their personal feelings about death. A total of 582 MOOC participants also completed this final activity, representing 18.68% of those who initially enrolled in the MOOC, and 43.11% of those who completed the three-words activity in the introductory week. This allowed a pre-post assessment of change in the sentiment of words describing death during exposure to the MOOC learning experience. Given the typical MOOC ‘funnel of participation’, the Dying2Learn MOOC-end retention rate for this final MOOC activity of interest in the current study can be considered above average in comparison to MOOCs in general [62–64].

The participants who completed the Time 1 activities in the introduction week of the course were predominately females (93.7%), living in Australia (87.7%), who identified as being a health professional (71.1%). Two-thirds of the sample held a university qualification (68.5%), and the average age of participants was 49.5 years (*sd* = 12.0), and ranged from 18 to 84 years of age. Even though the MOOC was designed for the general community, the course attracted a high proportion of health professionals (predominantly nurses) who were motivated to improve work-skills related to death and dying [65]. The demographic characteristics of our participating sample are typical of that usually found in the Australian health workforce population, of which about 90 percent are women, and the average age is in their 40s [66–68].

To assess the risk of participation bias in the study, the socio-demographic characteristics of the sample who completed the Time 1 word activities (*n* = 1350) were compared to the original sample of people who enrolled in the MOOC but did not subsequently participate in the introductory week activities (*n* = 1766). The sample retained was quite similar to the original sample of enrolees (no statistically significant differences on gender, university qualifications or health occupation), but the retained sample was statistically significantly older, and more likely to reside in Australia than those who enrolled but did not participate in the MOOC activities (small effect sizes of *Cohen’s d* = .215 and *d* = .296, respectively). Thus, the results of the present study may be somewhat less applicable to younger people and those residing outside Australia. An examination was also conducted to identify any differences between participants retained at Time 2 (*n* = 582) and those lost to attrition between Time 1 and Time 2 (*n* = 768) on socio-demographics and baseline word sentiment scores. No statistically significant differences were detected (*p* >.05 for all analyses). This suggests that the characteristics of the retained sample who completed the final activities was very similar to the Time 1 sample, and that sample attrition did not introduce undue bias. S1 Table provides further detail on these comparisons.

### Procedure

During enrolment, MOOC participants completed a short set of questions regarding their socio-demographic background and their general death attitudes. After completing enrolment, they were then able to access the first week of MOOC content. The Dying2Learn MOOC was developed within and hosted on the Open Learning Platform (https://www.openlearning.com/). The course content was developed by a team of academics with clinical knowledge and expertise in palliative care and online learning. It was designed to explore social issues around death and dying, with a focus on engaging people in conversations about death by posing questions and encouraging self-reflection. The MOOC content was delivered over 6 weeks, and included an introduction module, 4 core topic modules, and then a final reflections module in the last week. Module 1 focussed on how society engages with death through the language we use, humour, and mourning practices. Module 2 examined how death is portrayed in history, art, film, TV, and other media. Module 3 focussed on the role of medicine in how we die. Module 4 addressed death and its meaning in the internet age. Time required was estimated at 3 hours per module. During the introduction module, participants were asked to complete the ‘three-words’ activity by reporting words that describe their own feelings about death, and the feelings they perceive others to have. Approximately 6 weeks later, during the final reflections module, participants were asked again to complete the ‘three-words’ activity. Given the intention of the course to engender change in personal attitudes to death (and not necessarily change in perceptions of others’ attitudes), participants were only asked to report three words to describe their own personal feelings at the end of the course. Participants were instructed to simply go with their instinctual response. They were not explicitly given their original (Time 1) three-word choices, but they were not restricted from viewing it if they chose to. Following closure of the course, activity data was exported from the online learning platform, and was de-identified prior to analysis. The study methodology was approved by Flinders University Social and Behavioral Research Ethics Committee (Project No. 7247), and included the use of de-identified content from the MOOC activities for data analysis.

### Measures

#### Socio-demographic characteristics

Socio-demographic information was provided when participants enrolled in the MOOC, including their age in years, their gender (female/not), whether they self-identified as a health professional (yes/no), and highest level of completed education (some high school/completed high school/trade school or equivalent/university studies). Where possible, these questions were adapted from those used by the Australian Bureau of Statistics (ABS) [69, 70]. For analysis, we compared participants with university qualifications to those without. Finally, participants provided information about their location, and were classified as residing in Australia or residing outside Australia.

#### Words describing death

Words describing death were recorded in two MOOC activities. The first activity, during the introductory week, asked participants to ‘list three words that best describe how YOU feel about death and dying’. As a comparator, during this introductory activity participants were also asked to ‘list three words that you think best describe how ‘OTHERS’ in the general public feel about death and dying’. As an activity in the final reflections module at the end of the MOOC, participants were asked to again ‘list three words that best describe how YOU feel about death and dying’. Overall, a total of *n* = 10,246 words were listed, with *n* = 844 uniquely different words listed for personal feelings about death at Time 1, *n* = 542 unique words used at Time 2, and *n* = 611 unique words listed for others’ feelings about death.

#### Word sentiment scores

Word sentiment scores were assigned using Warriner and colleagues ‘ANEW_SUB’ lexicon of sentiment norms [37]. Participants’ exported responses from the three activities were first parsed, then all words were checked for spelling errors and converted to lowercase. Automatic text-based sentiment analysis of affective meaning from Warriner and colleagues’ [37] lexicon could then be used to distinguish three dimensions of emotions as defined by Osgood and colleagues [34] Theory of Emotions: valence (the pleasantness of a stimulus), arousal (the intensity of emotion provoked by a stimulus), and dominance (the degree of control exerted by a stimulus) [37]. Warriner and colleagues [37] provide a list of almost 14,000 lemma words that have been categorised on these three dimensions by around 1,800 respondents. Each dimension is scored on a scale from 1 to 9. The extracted three-word responses from each MOOC participant were cross-referenced to Warriner and colleagues’ [37] wordlist. Designed to advance the study of the interplay between language and emotion, this wordlist covers the word stock of the majority of well-known English language words quite substantially (representing approximately 25–50% of the words known to individuals), and provides a firm foundation for deriving the values of the remaining words [37]. Only lemmas (the base form of words) were included in the wordlist, because the emotional values of lemmas were expected to generalise to inflected forms. Because affective ratings are considered less useful for words unknown to most people, the words chosen for Warriner’s corpus were high-frequency words believed to be known by 70% or more of people who use the English language [71]. Participants in the present study were not restricted to choosing words from Warriner’s wordlist. If a word given by our sample was absent from Warriner’s wordlist, then it was first lemmatized and if that word was absent from Warriner’s wordlist, it was then stemmed and checked again against the wordlist. Lemmatisation reduces a word from its inflected forms (e.g., ‘sang’) to the root or base-form of the word (the one used as entries in dictionaries; e.g., ‘sing’). The process of stemming involves removing suffixes from the end of a word (e.g., ‘singing’ reduced to ‘sing’). Both lemmatisation and stemming were performed using the Standford CoreNLP, with stemming using the PorterStemmer algorithm [72]. The remaining unmatched words were then analysed by hand for a close match in Warriner and colleagues’ [37] wordlist. Where no close match was able to be found, the word was coded as missing.

Across the three activities, there was a total of 10,246 words listed by participants (there were *n* = 278 word-slots that were left blank by participants). For most words (*n* = 8602 or 83.95% of words) we were able to assign sentiment scores to the original word used by the participant. For 808 words (7.89%), the lemma of the original word was used, and for 69 words (0.69%) the stem of the original word was used. For words without an original, stem or lemma match, two researchers manually searched Warriner’s dictionary for an alternative stem/lemma that was missed by automated processes. Chosen word alternatives were cross-verified by two raters prior to being assigned. We were unable to assign a Warriner’s score to 134 words due to no logical lemma or stem alternative being available in the Warriner’s dictionary, or because the lemma/stem could have multiple meanings (e.g., ‘after’ was not replaced with ‘afterlife’ from Warriner’s wordlist, because ‘after’ may have been referring to ‘later’). Thus overall, a total of 10,112 (98.69%) of the words provided were able to be assigned sentiment scores from Warriner’s wordlist. (For details, see S2 Table.)

Three types of ratings from Warriner’s corpus were assigned to each word, in line with Osgood, Suci, and Tannenbaum’s [34] theory of emotions. The first (and most important) type of rating concerns the valence (or pleasantness) of the emotions invoked by a word, ranging from 1 ‘happy’ to 9 ‘unhappy’. The second dimension addresses the degree of arousal evoked by a word, ranging from 1 ‘excited’ to 9 ‘calm’. The third dimension refers to the dominance/power of the word (the extent to which the word denotes something that is weak/submissive or strong/dominant), and ranges from 1 ‘controlled externally’ to 9 ‘in control’. The valence and arousal rating scores were reversed post-hoc to maintain a more intuitive low-to-high scale (e.g., sad to happy rather than happy to sad) across all three dimensions. Valence, arousal, and dominance each represent unique linguistic aspects of words, and are best understood as a three-dimensional concept, rather than a construct that can be treated as a summed composite [34]. Indeed, non-linear associations have been found between the three dimensions [37]. Therefore, each word provided by a participant was assigned three scores from Warriner’s database–one for valence, one for arousal, and one for dominance. Based on the 13,915 words rated in Warriner’s wordlist, the available range on each dimension within Warriner’s word dictionary was 1.26 to 8.53 for valence, 1.60 to 7.79 for arousal, and 1.68 to 7.90 for dominance.

A final score on valence on Time 1 personal feelings about death was derived by averaging the valence scores of the three words provided in the introductory module. The same averaging process was used to arrive at a final arousal score and final dominance score for personal words at Time 1. The three Time 1 ‘others’ words, and the three Time 2 personal words were also averaged in the same manner. This process resulted in nine variables for data analysis, three for each activity: Time 1 personal words valence score, Time 1 personal words arousal score, Time 1 personal words dominance score, Time 1 others’ words valence score, Time 1 others’ words arousal score, Time 1 others’ words dominance score, Time 2 personal words valence score, Time 2 personal words arousal score, and Time 2 personal words dominance score.

### Statistical approach

The key objectives of this study were to (a) examine whether the sentiment of words people used to describe their personal feelings about death differed from their perceptions of the feelings of other people; and (b) to examine whether the sentiment of words people used to describe their personal feelings about death changed over a period of time during which they were participating in an online course about death. This study analyses data based on enrolment in the MOOC, and responses provided for the three-words activities in the introductory week (Time 1) and responses to this activity when repeated in the final week of the MOOC (Time 2). Given sentiment analyses generate three different scores (valence, arousal, dominance dimensions) for each activity, we used a more conservative statistical significance level of *p* < .0166 (Bonferroni correction: .05/3 = .0166) to adjust for multiple testing and account for the increased risk of Type I error [73]. As recommended [74], we also calculated effect sizes to consider the magnitude of the effects found, which were interpreted using standard recommendations [75, 76].

An a priori power analysis was conducted using G*Power 3.1.9.7 [77] to test the difference between two dependent means using a two-tailed test, a very small effect size (*d* = .15), and an *p* level of .0166. Results showed that a total sample of *n* = 729 participants was required to achieve 95% power. Data analyses were conducted in IBM SPSS Version 23.

First, we provided a descriptive analysis of the actual types of words used by participants and their frequency of use, and the descriptive statistics on sentiment scores. Scores on the sentiment variables did not significantly deviate from normality.

S1 Table outlines the patterns of missing data and plausible bias due to dropout. The prevalence of missing data in the dataset was examined, with sensitivity analyses undertaken (See S1 File). Missing Values Analysis indicated a proportion of participants completed some but not all of the 3 word-slot responses for each of the three activities, with the major point of attrition occurring between Time 1 and Time 2 (65% missing data). Multiple Imputation (MI) was conducted using fully conditional specification (FCS) Markov chain Monte Carlo (MCMC) method in SPSS to assess the impact of the missing values. The number of imputations was set to 20 and the maximum iteration to 1000 [78]. A sensitivity analysis was undertaken. In line with recommendations [79, 80], we conducted complete-case data analysis in parallel to data analysis utilising the MI pooled function, and report complete-case results alongside the MI results in S1 File. MI pooled results showed that the means and standard errors were similar to those found when utilising listwise deletion (complete-cases), and the overall conclusions were the same, but the effect sizes were slightly smaller when using MI data. A simulation study [79] performed to examine the effectiveness of MI with various levels of missingness found multiple instances where MI was effective at 75% missingness, detecting slightly less bias when using MI pooled data compared to complete-cases. Therefore, in this paper we have reported results based on MI pooled data (n = 1491) [79, 80].

Bivariate associations between socio-demographic variables and the sentiment score variables from each activity were examined using Independent Samples T-test and Pearson’s correlations. Cross-sectional analyses of Time 1 average sentiment scores on valence, arousal and dominance for self and others were compared within-samples using Paired-Samples T-test, comparing scores on personal perspective versus score on perspective of others in the general public. This analysis is appropriate because of its within-subjects nature and the scores on sentiment variables for self and others were measured on the same scale [73].

Longitudinal analyses examining pre-to-post changes in personal word valence, arousal, and dominance scores over the course of the MOOC were conducted using repeated-measures Paired-Samples T-test, comparing scores at the two time-points (measured MOOC-start and MOOC-end). Longitudinally, a series of Hierarchical Multiple Linear Regressions were used to identify socio-demographic variables predictive of valence, arousal and dominance sentiment scores at the end of the MOOC. By adjusting for scores on sentiment at baseline, we examined the socio-demographic predictors of change over time in word sentiment scores pre-to-post MOOC. It provided an indicator of whether specific groups were more likely to change in word sentiment during in the time-period of their participation in the MOOC.

## Results

### Descriptive analyses

#### Frequently-reported words in each activity

Of the *n* = 4259 words provided for the baseline personal death words activity, *n* = 844 were unique words. For the baseline others’ words activity, *n* = 611 of the *n* = 4128 words given were unique. At MOOC-end when participants provided personal death words again, *n* = 542 of the *n* = 1859 words given were unique. Table 1 outlines the 20 most commonly-reported words for the three activities. It can be seen that the words chosen for the general public (e.g., fear, sad, scary) were more negative than those chosen to represent their own personal feelings (e.g., inevitable, sad, peace). Comparing the words given at baseline by participants, 20.7% of people mentioned ‘inevitable’ as a word for their own feelings, compared to 6.4% who mentioned this word when describing other people’s feelings. At baseline 3.9% mentioned ‘scared’ for their own feelings, but 11.3% mentioned ‘scared’ for other’s feelings. Similarly, 3.7% mentioned ‘fear’ for their own feelings, versus 21.4% who said this for other’s feelings. In respect to differences at the beginning and ending of the MOOC on personal feelings, the proportion of participants who said ‘peaceful’ went from 8.7% to 14.8%, and the proportion who said ‘sad’ went from 14.2% to 7.9%. ‘Inevitable’, ‘final’ and ‘peace’ were mentioned by similar proportions of participants at each time-point.

**Table 1:** Most frequently-reported words for describing feelings about death.

20 Most Common Words	Baseline Personal Words (Unique Words Given n = 844)		Baseline Others’ Words (Unique Words Given n = 611)		MOOC-End Personal Words (Unique Words Given n = 542)	
	*Word*	*n Count*^*a*^ *(%*^*b*^*)*	*Word*	*n Count*^*a*^ *(%*^*b*^*)*	*Word*	*n Count*^*a*^ *(%*^*b*^*)*
**1**	inevitable	280 (20.7%)	fear	289 (21.4%)	inevitable	116 (19.9%)
**2**	sad	192 (14.2%)	sad	251 (18.6%)	peaceful	86 (14.9%)
**3**	peace	143 (10.6%)	scary	178 (13.2%)	peace	63 (10.8%)
**4**	natural	135 (10.0%)	loss	173 (12.8%)	natural	51 (8.8%)
**5**	peaceful	118 (8.7%)	scared	153 (11.3%)	sad	46 (7.9%)
**6**	final	105 (7.8%)	sadness	114 (8.4%)	final	40 (6.9%)
**7**	loss	103 (7.6%)	fearful	108 (8.0%)	comfortable	37 (6.4%)
**8**	sadness	99 (7.3%)	grief	105 (7.8%)	accepting	32 (5.5%)
**9**	unknown	77 (5.7%)	unknown	101 (7.5%)	acceptance	30 (5.2%)
**10**	release	70 (5.2%)	denial	93 (6.9%)	calm	22 (3.8%)
**11**	relief	63 (4.7%)	taboo	90 (6.7%)	curious	22 (3.8%)
**12**	scared	52 (3.9%)	pain	88 (6.5%)	sadness	22 (3.8%)
**13**	curious	51 (3.8%)	inevitable	87 (6.4%)	informed	21 (3.6%)
**14**	emotional	51 (3.8%)	painful	81 (6.0%)	normal	21 (3.6%)
**15**	comfortable	50 (3.7%)	frightening	69 (5.1%)	loss	20 (3.4%)
**16**	grief	50 (3.7%)	final	62 (4.6%)	love	20 (3.4%)
**17**	fear	50 (3.7%)	end	52 (3.9%)	transition	20 (3.4%)
**18**	normal	47 (3.5%)	lonely	49 (3.6%)	dignity	19 (3.3%)
**19**	accepting	44 (3.3%)	frightened	41 (3.0%)	life	19 (3.3%)
**20**	scary	44 (3.3%)	afraid	39 (2.9%)	painfree	18 (3.1%)

*Notes*.

^*a*.^ Word frequencies by word, not participant.

^*b*.^ Percentages are calculated as a proportion of the total number of participants who listed that word in their response to the activity (n = 1350 participants for baseline activities; and n = 582 for MOOC-End activity).

Sixteen of the top 20 words chosen to represent perceptions of others’ feelings had clearly apparent negative connotations, compared to 8 of the top 20 words chosen for their own feelings at baseline (80% vs 40%). Further, the types of words participants chose to describe their feelings at the end of the MOOC (e.g., inevitable, peaceful, peace) tended to be less negative than those chosen at the beginning, and more along the lines of acceptance and comfort. Only 3 of the top 20 words chosen at the end of the MOOC had clearly apparent negative connotations (40% vs 15%) This assessment of the inherent connotations of the words is consistent with the valence, arousal, and dominance sentiment scores applied to these words [37]. The word ‘inevitable’ is assigned Warriner scores of valence = 4.10, arousal = 4.27, and dominance = 3.15. This compares to the word ‘sad’ (valence = 2.10; arousal = 3.49; dominance = 3.84), the word ‘fear’ (valence = 2.93; arousal = 6.14; dominance = 3.32), and ‘scary’ (valence = 3.00; arousal = 5.35; dominance = 3.70), versus the word ‘peaceful’ (valence = 8.0; arousal = 4.38; dominance = 6.84), ‘peace’ (valence = 7.75; arousal = 4.65; dominance = 7.17), and ‘natural’ (valence = 6.42; arousal = 3.67; dominance = 5.16). As a point of comparison, in Warriner’s corpus the actual word ‘death’ is assigned sentiment scores of 1.89 for valence, 5.53 for arousal, and 3.42 for dominance, and the actual word ‘die’ is assigned sentiment scores of 1.67 for valence, 6.90 for arousal, and 3.29 for dominance.

#### Frequency of word differences across activities

A key point of interest is whether people chose different words for each activity, or tended to re-report the same ones (See S3 Table). Usually, all three words that participants chose in each scenario were different. At baseline when participants chose words for their own feelings towards death (Activity 1) and their perspective of how others feel about death (Activity 2), in 69.5% of cases all 3 words chosen were different. At least one word was different in 96.4% of cases, and in only 3.6% of cases did a person provide the exact same 3 words for themselves and for others. This suggested there was a perceived discrepancy between one’s own feelings about death and the feelings of others. The words chosen to describe how they personally feel about death between the baseline activity and the activity at the end of the course showed that there was a number of differences in the words chosen. Participants chose three completely different words in 52.5% of cases, at least one different word in 95.4% cases, and in only 4.6% of cases did all 3 words chosen at time 2 remain the same as those chosen at baseline. Thus, by the end of the MOOC, the words chosen to express their feelings about death changed for most participants.

#### Sentiment score descriptive statistics and associations with socio-demographic characteristics

In Warriner’s corpus of 13,915 words [37], the mean scores for Valence were *m* = 5.06 (*SD* = 1.68); for Arousal *m* = 4.21 (*SD* = 2.30), and for Dominance *m* = 5.18 (*SD* = 2.16), respectively. This compares to our average means at baseline (prior to the MOOC educational intervention) of *m* = 4.42 (*SD* = 1.42) for valence, *m* = 4.57 (*SD* = 0.61) for arousal, and *m* = 4.64 (*SD* = 0.94) for dominance. This indicates that the sentiment of words chosen specifically to represent the concept of death in our study were unhappier, more arousing, and more submissive/controlled-externally than the sentiment averages found for all 13,915 words in Warriner’s normative wordlist. Thus, the sentiment of words used specifically in consideration of death was more negative than those representing the spectrum of sentiment for well-known lemma words in the English language (based on Warriner’s corpus). The score distributions on valence, arousal, and dominance, with example words at the peaks of the distributions, are shown in S1–S3 Figs, respectively. Descriptive statistics on sentiment scores for each activity individually are shown in Tables 2 and 3.

**Table 2:** Paired-Samples t-test results: Word sentiment scores for self personally and for the general public, *n* = 1491.

Outcome	*n* ^d^	Personal perspective *Mean (SE); 95% CI*	Public perspective *Mean (SE); 95% CI*	*Mean Diff*. *(SE); 95% CI*	Perspective effects		
					*t (df)*	*p*	*Cohen’s d*
Word Valence Score ^a^	1491	5.25 (0.0351);	3.56 (0.026);	1.695 (0.0417);	40.67 (1882)	< .0005	-1.048
		5.18 to 5.32	4.51 to 3.61	1.61 to 1.77			
Word Arousal Score ^b^	1491	4.33 (0.015);	4.82 (0.015);	-0.484 (0.0210);	-23.03 (1085)	< .0005	0.600
		4.31 to 4.36	4.79 to 4.85	-0.5250 to -0.4430			
Word Dominance Score ^c^	1491	5.16 (0.025);	4.12 (0.017);	1.026 (0.0287);	35.74 (4423)	< .0005	-0.907
		5.10 to 5.19	4.06 to 4.15	0.9700 to 1.0830			

*Notes*.

^a^ To contextualise these valence sentiment score differences, words reported in our study that are closest to the mean scores for Valence were: 5.25 = ‘metamorphosis’; ‘silence’, versus 3.56 = ‘nervous’; ‘reticent’.

^b ^To contextualise these arousal sentiment score differences, words reported in our study that are closest to the mean scores for Arousal were: 4.33 = ‘intimidating’; ‘doubt’, versus 4.82 = ‘denial’; ‘blame’.

^c ^To contextualise these dominance sentiment score differences, words reported in our study that are closest to the mean scores for Dominance were: 5.16 = ‘natural’; ‘tentative’, versus 4.11 = ‘punishment’; ‘sorrowful’.

^d^ Analyses conducted using MI imputed Pooled data, *n* = 1491. For parallel analyses conducted using *n* = 1350 complete cases, see S1 File.

**Table 3:** Paired-Samples t-test results: Personal words sentiment scores at baseline and MOOC-end, *n* = 1491.

Outcome	*n* ^d^	*Baseline Mean (SE); 95% CI*	*MOOC-End Mean (SE); 95% CI*	*Mean Diff*. *(SE); 95% CI*	Time effects		
					*t (df)*	*P*	*Cohen’s d*
Word Valence Score ^a^	1491	5.25 (0.035);	5.90 (0.038);	-0.652 (0.04541)	-14.36 (100)	< .0005	0.408
		5.183 to 5.321	5.82 to 5.98	-0.7418 to -0.5622			
Word Arousal Score ^b^	1491	4.34 (0.015);	4.19 (0.023);	0.148 (0.02579)	5.74 (68)	< .0005	-0.155
		4.30 to 4.37	4.14 to 4.23	0.0966 to 0.1991			
Word Dominance Score ^c^	1491	5.15 (0.025);	5.63 (0.037);	-0.488 (0.04111)	-11.87 (53)	< .0005	-0.339
		5.11 to 5.20	5.56 to 5.70	-0.5670 to -0.4060			

*Notes*.

^a ^To contextualise these valence sentiment score differences over time, words reported in our study that are closest to the mean scores for Valence were: 5.25 = ‘silence’; ‘imminent’, versus 5.90 = ‘resonance’; ‘realistic’.

^b ^To contextualise these arousal sentiment score differences over time, words reported in our study that are closest to the mean scores for Arousal were: 4.34 = ‘solace’; ‘doubt’, versus 4.19 = ‘memories’; ‘optimistic’.

^c^ To contextualise these dominance sentiment score differences over time, words reported in our study that are closest to the mean scores for Dominance were: 5.15 = ‘anger’; tentative’, versus 5.63 = ‘regret’; ‘relief’.

^d^ Analyses conducted using MI imputed Pooled data, *n* = 1491. For parallel analyses conducted using *n* = 582 complete cases, see S1 File.

Bivariate relationships between socio-demographic characteristics and word sentiment scores for the three activities were investigated (See S4 Table). Compared to participants residing in Australia, participants residing outside of Australia had significantly higher baseline arousal scores (*m* = 4.32 [*SE* = .02] vs *m* = 4.44 [*SE* = .04]) for words chosen to represent their personal feelings about death. Participants who were health professionals only differed from other participants on one sentiment score, with health professionals’ baseline personal arousal score (*m* = 4.30, *SE* = .02) significantly lower than those who were not health professionals (*m* = 4.41, *SE* = .03). Age was associated with the sentiment of words chosen to represent personal feelings towards death at baseline, with older age demonstrating a small significant positive association with higher scores on baseline personal valence. University education was only influential on participants perceptions of how other people feel about death. Compared to participants without a university education, participants with a university qualification obtained significantly lower valence scores (*m* = 3.65 [*SE* = .05] vs *m* = 3.51 [*SE* = .03]) and dominance scores (*m* = 4.19 [*SE* = .03] vs *m* = 4.08 [*SE* = .02]) for the words they chose to represent their perception of how other people feel about death (differences reported significant at *p* < .0166; all effects were small). There was little influence of socio-demographic characteristics on the sentiment of words chosen at the end of the MOOC. There were no other significant relationships found between socio-demographic characteristics and word sentiment scores. See S4 Table.

### Sentiment of words for self as compared to others

Table 2 reports a series of paired-samples t-tests (within-subjects) comparing scores on perspectives regarding personal word sentiment at baseline versus word sentiment attributed to others in the general public. MOOC participants’ chosen words to describe their own personal feelings about death had significantly more positive sentiment scores than the words they used to describe how they think/perceive other people in the general public feel (all *p* < .0005). Compared to personal words chosen to describe their own feelings, words chosen to represent others in the general public had a substantially lower valence, higher arousal, and lower dominance. The *Cohen’s d* statistics ranged from .60 to -1.05, indicating large effect sizes on valence and dominance scores, and a moderate effect size on arousal scores.

Examination of the means indicate that valence scores were higher for words attributed to one’s own feelings about death than those attributed to the feelings of others (a difference of 1.70). Arousal scores were lower for words expressing personal feelings than they were for words attributed to others’ feelings (difference of 0.48). Dominance scores were higher for words attributed to personal feelings than words representing perceptions of others’ feelings (difference of 1.03). The absolute score differences may appear small, but examination of the actual words reported in our study that correspond with the mean scores can provide contextualisation. The corresponding words for valence means were ‘metamorphosis’ and ‘silence’, for personal feelings versus ‘nervous’ and ‘reticent’ for others’ feelings; for arousal means were ‘intimidating’ and ‘doubt’, versus ‘denial’ and ‘blame’; and for dominance means were ‘natural’ and ‘tentative’ versus ‘punishment’ and ‘sorrowful’.

### Changes in personal word sentiment from baseline to MOOC-end

Table 3 shows the result of a series of paired-samples t-tests comparing scores over time on personal word sentiment at baseline and MOOC-end. MOOC participants’ chosen words to describe their own feelings about death overall showed significantly more positive sentiment at the end of the MOOC course than they did at the beginning (all *p* < .0005). The *Cohen’s d* statistics for ranged from -.155 to .408, indicating small-to-moderate effect sizes.

The means indicate that valence scores for words describing personal feelings towards death increased over time from the beginning to the end of the online course (0.65 score increase, or 12.4% increase), and dominance scores for words describing one’s own feelings also increased by the end of the MOOC (0.49 score increase, or 9.5% increase; ). There was a small reduction in arousal scores for personal feelings about death by the end of the MOOC (0.15 score decrease, or 3.5% reduction). The corresponding words for valence means were ‘silence’ and ‘imminent’ at baseline versus ‘resonance’ and ‘realistic’ at MOOC-end; for arousal means were ‘solace’ and ‘doubt’, versus ‘memories’ and ‘optimistic’; and for dominance means were ‘anger’ and ‘tentative’ versus ‘relief’ and ‘regret’.

**Fig 1:**
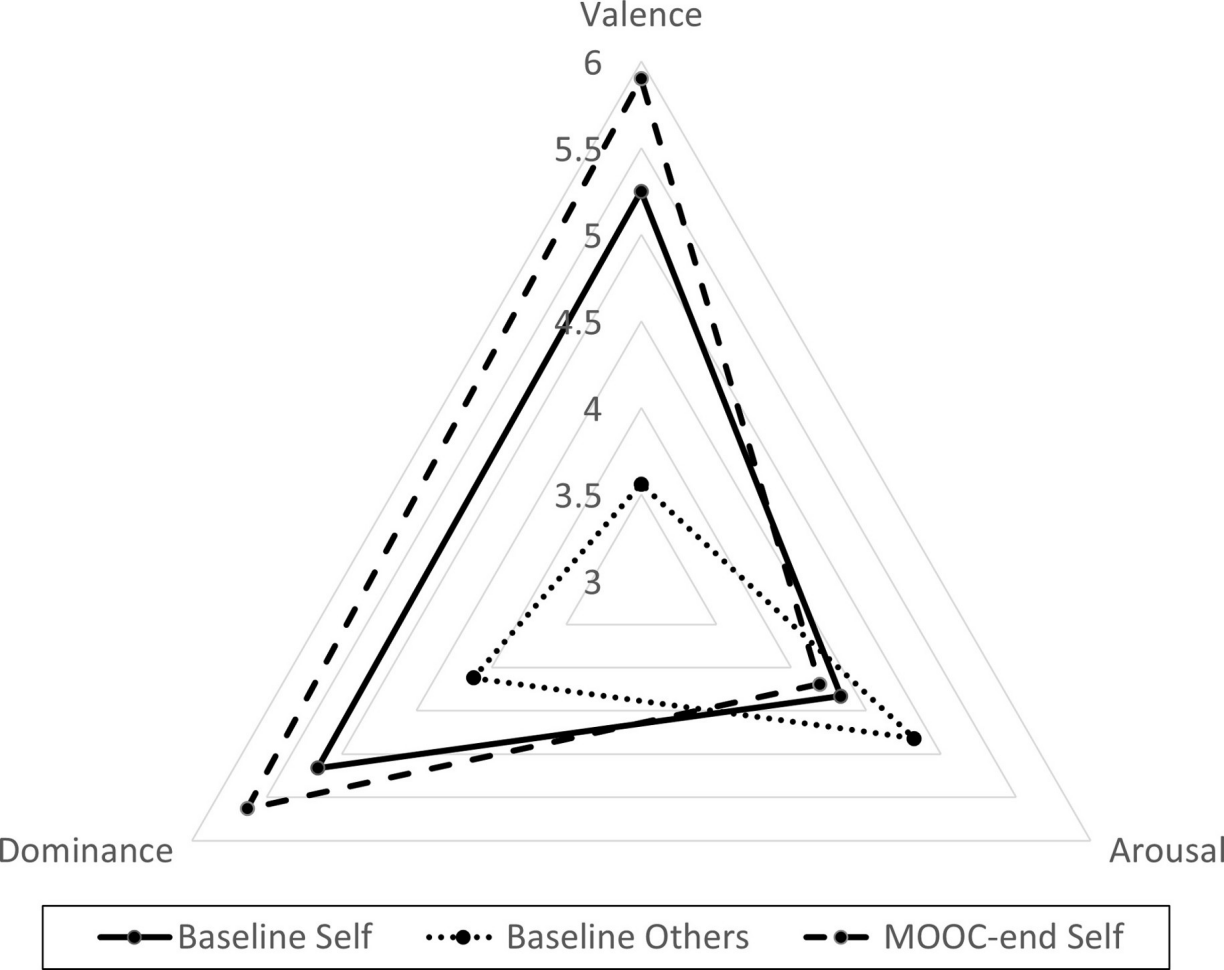
Radar plot comparing differences in death word sentiment dimensions between baseline self and others, and baseline self and MOOC-end. Solid line represents Baseline Self; dotted line represents Baseline Others; dashed line represents MOOC-End Self.

A combined visual representation of the valence, arousal, and dominance sentiment scores across the three different activities is shown with a radar plot in Fig 1. It indicates that the words given to represent perceptions of others’ feelings about death were more arousing, less valent and less dominant when compared to the words used to describe one’s own feelings about death at baseline and MOOC-end. The words chosen to describe one’s own feelings at the end of the MOOC had higher valence and higher dominance than those chosen at the start of the course, and were slightly less arousing.

Table 4 examines socio-demographic predictors of change over time in personal word sentiment scores, by using hierarchical linear regressions to predict scores on personal word sentiment at the completion of the MOOC after adjusting for personal word sentiment scores at baseline. This provides an indicator of whether specific demographic groups were more or less likely to change in personal sentiment scores during the time-period participating in the MOOC. The results of the three regression models are shown in Table 4 for word valence, word arousal, and word dominance outcome scores, respectively. For all three aspects of word sentiment, baseline levels on these sentiment scores were strong predictors of the subsequent sentiment score on valence, arousal and dominance at the end of the MOOC (all *p* < .0005; each making moderately-sized unique contributions to the variance explained; pooled Semipartial Correlation Coefficients of .363, .194. and .304, respectively). The final models accounted for an average of 14.75%, 4.47%, and 11.22% of the variance in MOOC-end valence, arousal, and dominance sentiment scores, respectively. In the final model, there were no significant unique effects of location, occupation, or education on the level of change in word sentiment scores over time, suggesting changes in word sentiment occurred similarly for participants regardless of their location, occupation or education. After taking into account baseline levels of sentiment, the only variable suggesting significant unique associations with the change in word sentiment scores over time was participant age. Change in arousal scores were not influenced by age. However, after adjusting for all other factors in the model, participants who were younger showed significantly more positive change in valence scores by the end of the MOOC, and to a lesser extent also for dominance (both made small unique contributions to the variance explained; pooled Semipartial Correlation Coefficients of -.088 and -.080, respectively). This meant that the older the participant, the less change there was in their dominance and valence scores. Post-hoc analyses comparing the mean change scores of age groups confirmed this, indicating that younger participants (under 40) experienced significantly more improvement in dominance (*t* = 2.81, *df* = 85.45, *p* < .006) and valence (*t* = 2.84, *df* = 87.76 *p* < .006) scores over time (i.e., became more in-control/dominant and more positive/pleasant) than older participants aged 40+. The increase in mean dominance scores and valence scores over time for participants under age 40 (*m* = 0.69 and *m* = 0.91) was larger than the increase seen for participants aged 40+ (*m* = 0.43 and *m* = 0.58), though the size of the effects were small.

**Table 4:** Imputed Hierarchical Multiple Linear Regressions predicting MOOC-end sentiment scores, adjusting for baseline sentiment scores (*n* = 1491)^a^.

	MOOC-End Sentiment Scores								
	Word Valence Score			Word Arousal Score			Word Dominance Score		
Baseline Predictor Variables	*Unstandardised B (SE)*	*B 95%CI*	*p*	*Unstandardised B (SE)*	*B 95%CI*	*p*	*Unstandardised B (SE)*	*B 95%CI*	*p*
***Initial Model Variables*:**									
**Baseline Sentiment score**	.313 (.036)	.241 to .385	< .0005	.193 (.046)	.099 to .287	< .0005	.302 (.042)	.218 to .386	< .0005
***Final Model Variables*:**									
**Baseline Sentiment score**	.317 (.035)	.246 to .389	< .0005	.196 (.047)	.101 to .291	< .0005	.302 (.041)	.220 to .384	< .0005
**Age**	-.008 (.003)	-.015 to -.002	.015	.002 (.002)	-.002 to .005	.319	-.006 (.003)	-.012 to -.001	.024
**Located in Australia (Yes)**	-.171 (.138)	-.449 to .107	.233	.005 (.059)	-.113 to .123	.929	-.184 (.117)	-.420 to .052	.124
**Health Professional (Yes)**	.114 (.106)	-.100 to .329	.290	.011 (.051)	-.091 to .113	.832	.109 (.088)	-.069 to .286	.224
**University Education (Yes)**	.017 (.098)	-.181 to .214	.865	.080 (.050)	-.021 to .180	.118	-.025 (.081)	-.187 to .138	.761
***Model Fit Statistics (Ranges in 20 imputed datasets)*** ^***a***^**:**									
***Initial R***^***2***^ ***range***	.094 to .201			.013 to .068			.058 to .125		
***F range***	153.23 to 373.22 (all *p* < .0005)			19.47 to 108.71 (all *p* < .0005)			91.11 to 211.27 (all *p* < .0005)		
***ΔR***^***2***^ ***range***	.005 to .028			.001 to .019			.003 to .030		
***ΔF range***	2.17 to 12.26 (*p* = .000 to 007)			0.50 to 7.31 (*p* = .000 to .733)			1.20 to 12.92 (*p* = .000 to .311)		
***Final Model p range***	All < .0005			All < .0005			All < .0005		

*Notes*.

^a^ Pooled estimates of Model fit are not provided in SPSS. So we have reported the range of model fit statistics obtained in the 20 imputed datasets.

## Discussion

### Summary of findings

Understanding public attitudes and feelings towards death is needed in order to inform health policies [3]. This study provided the first description of emotive attitudes of people asked to consider death utilising sentiment analysis. Applying multidisciplinary methodologies to a novel activity gave a deeper understanding of affective death attitudes in a community sample. We analysed the emotional sentiment of words people chose to describe feelings about death, for themselves, for perceptions of others, and for changes over the time-period during exposure to a course about death. The results demonstrated that text-sentiment analyses can provide a meaningful approach to death attitude research, consistent with previous linguistic investigations [20–22, 29]. ‘Sad’ was a word that was prevalent throughout, regardless of whether referring to feelings for oneself or for others, or feelings captured at the beginning or ending of a course about death and dying. Thus ‘sad’ feelings were universally linked to death. Nonetheless, the MOOC participants commonly chose words to express their perception of other’s feelings towards death that were considerably more emotionally negative (‘fear’, ‘scary’, ‘loss’) than the words chosen to express their own feelings towards death (‘inevitable’, ‘peace’, ‘natural’). When we moved beyond the self-report of words themselves and applied numeric sentiment scores to the words chosen, we found that the sentiment expressed to represent one’s own perspective was significantly more positive compared to those representing the perceived perspective of others. The words chosen to represent the feelings of others indicated sentiment that was more unhappy/unpleasant, more arousing/excitable, and more submissive/dominated by external forces. This aligns with findings from other studies [5, 59, 60]. Our findings leave us with the question of why the MOOC participants think that others feel so differently about death than they do themselves? Part of the explanation is likely to lay in the self-selected nature of our sample of people who chose to participate in a course about death, a considerable proportion of whom identified as health professionals and may have seen themselves as more informed and conditioned to death. But, the sentiment of the words chosen to represent the perceived feelings of others does demonstrate the assumptions people make about other peoples’ negative reactions regarding death. It is possible that these assumptions have implications for the willingness of people to start conversations about death and dying with the people around them. If we perceive that others will become distressed by bringing up the topic of death, are we less likely to attempt raising the topic? Does this avoidance then leave important things unsaid?

When we compared the types of words participants chose to describe their personal feelings about death at the beginning and the end of the MOOC, we found that the most frequently mentioned words were quite similar at both time points (e.g., inevitable, peaceful, peace, natural, sad), but that mentions of more negative words like ‘sad’ reduced considerably in frequency over time, along with a corresponding increase in mentions of words related to acceptance and comfort. The specific words chosen by individuals at each time point showed more changes over time than stability. When the sentiment of the words chosen to express personal feelings about death at both time-points was analysed, we found that participants chose happier valence words, less arousing words, and more dominant words at the end of the course about death than they did at the beginning. This finding suggests that participating in an online course about death and dying may have had a positive effect on the type of language people chose to express their feelings about death, with the emotional sentiment of the language used by the end of the course subsequently becoming more pleasant/positive, calmer, and more internally controlled. Given that the sentiment of words deals with emotion in relation to an issue, this suggests that over time the course may has assisted participants in becoming more emotionally accepting in their feelings and attitude towards death. It is also important to note therefore that spending time thinking and learning about death over a five-week period did not appear to have a negative emotional impact on course participants. This finding is consistent with our findings from research with our 2016 cohort using formal scale measures [6] and evaluation questionnaires [5] geared more towards behavioural indicators. The findings of the present study indicate that participation in the MOOC potentially had a positive emotional influence on the participants, in addition to the behavioural and cognitive influence demonstrated in earlier studies [5, 6]. This offers valuable triangulating evidence validating the potentially beneficial effects of participating in an online course about death.

Our multivariate findings demonstrated that the positive changes in sentiment occurred similarly for participants regardless of education, occupation, and location. However, an interesting influence of age was found on the rate of change in valence and dominance sentiment scores from the beginning to the end of the course. From the outset, younger participants scored lower on death sentiment valence and dominance, and compared to older participants, younger participants experienced greater change during course participation on the sentiment of the language chosen to express personal feelings about death, with greater increases in the pleasantness (valence) and dominance (power and control) in the words they chose at the end of the course. This may be in part due to younger participants having lower valence and dominance scores at baseline, meaning there was more opportunity for improvement in their scores by the end of the course. For older participants, the influence of a course discussing death is perhaps less due to having more personal exposure and proximity to death in their life and thus greater mortality salience. Therefore, it’s possible that participation in an online course about death may be especially beneficial for younger people, potentially assisting them to become less emotionally negative and more accepting of death and its inevitability.

### Implications

The results from this study provide further validation of the emotive nature of death and dying. It provided quantification of the strength and direction of affective responses to death and the differential perceptions of death attitudes participants applied to others in the community. Word sentiment analysis was a useful adjunct to traditional self-reports, and is arguably less invasive.

The words and labels used in clinical encounters can have differing effects upon patients and clinicians. For example, Tayler and Ogden [81] reported the tendency of doctors to use euphemisms instead of the direct term ‘heart failure’ due to the dilemma of the latter causing more negative emotional reactions in patients. On the other hand, a study of more benign conditions (gastroenteritis/tonsillitis) found the use of clinical language over lay language can validate the patient’s sick role and can increase confidence in the doctor [82]. The use of euphemisms to avoid the word death is reportedly common, and accompanied by the potential for misunderstandings [20, 22, 58]. Thus, words are not neutral. They can have an impact on patients and people–they can bring people with us or alienate them, they can be interpreted differently by people, they can be delivered in personal or impersonal ways, and they can sway a person’s actions. There may be implications worth exploring for clinical settings, particularly for awareness of the choice of words verbalised in clinical encounters related to the end-of-life. Understanding the sentiment of words provides valuable insight into the emotional connotations tied to words we use, and could be useful for guiding clinical conversations in palliative care [20, 22].

The knowledge gained from this study about community death attitudes could potentially inform the creation of dialogue and messaging used in public health campaigns about the end-of-life preparedness. The use of higher valence and dominance words might bolster the persuasiveness of campaigns in a way that potentially leads to more effective emotional engagement and behavioural activation in target populations [37, 83].

### Strengths, limitations, and future directions

Whilst this study was based on a large sample, several potential limitations require consideration. Like most MOOC samples, our sample is unlikely to be representative of the general population [62–64]. Participants were a self-selected sample of people in the community who chose to enrol in a course about death, and therefore may have been more likely to feel comfortable with the topic of death from the outset than people in the general population. Our sample also included a considerable proportion of people who identified as health professionals, which may impact on death attitudes given their greater opportunity for exposure and possibility of more desensitised death-related emotions. The sample was also comprised of a vast majority of females, which meant that gender comparisons could not be meaningfully undertaken. Furthermore, the results of the present study may be somewhat less representative of younger people and those residing outside Australia, because enrolees who subsequently went on to participate in the MOOC activities were slightly older and more likely to be located in Australia than those who enrolled but did not participate. Replication of the study with a representative community sample would be a valuable avenue for future research, to garner a stronger understanding of socio-demographic variations in the sentiment of words about death. Comparing death word sentiment for various age cohorts in the community with differing death exposure experiences is particularly needed, as well as direct dyad comparisons of self-other death attitude perceptions. It would also be informative to examine how respondents’ death word sentiment relates to their self-reported fear of death and other emotive death attitudes rated on standardised instruments. This could enable a broader insight into various aspects people associate with death [7].

A strength of this study was the inclusion of a longitudinal component that observed changes over time in the sentiment of words chosen to express feelings about death. However, course attrition meant that slightly less than half of participants that provided words at baseline also provided data at the end of the course. It is possible that those participants who were less inclined to complete the Time 2 activity were also less likely to have experienced a positive emotional response to the course. Nonetheless sensitivity analyses using alternative approaches (Multiple Imputation) to account for the missing data obtained similar results and conclusions to those using the complete-case sample. In the final week of the course when participants were asked to provide three death words again, it was possible for them to go back and look at their previous word choices from the beginning of the course. It is not clear how many participants went back and checked their first response, versus those who followed instructions to simply go with their instinctual response. It is not known what effect any comparative check by participants may have had on the words they chose to report at Time 2.

Of further importance is that our longitudinal assessment of change over time did not include a comparison group of people who were not exposed to the MOOC. Without this comparison group, it is impossible to rule out alternative explanations for the reasons word sentiment scores changed from the start to the end of the course. Other factors occurring within lives of participants while completing the MOOC may have caused changes on death sentiment over time that were not related to MOOC completion. Future research including a control group is warranted.

In this study, we applied sentiment analysis to three specifically chosen content words rather than to a written sentence, or a more generalised analysis of written text. However, it is possible that by giving participants explicit instructions we obtained more expressive emotional content words than we would have if a sentence was requested, given that typically only a small proportion of words used are classified as emotive [14]. The results may have differed if the activity used a sentence instead, but it may have reduced the emotive data elements provided, being more likely to have been limited to one core theme/lemma. Alternatively, utilising a much larger textual response may have provided a deeper understanding of emotions, as there is evidence that non-content function words such as prepositions and pronouns can also convey affective content [84, 85]. It would be worthwhile to explore the use of these different methodologies in future research, such as examining the effect of the online course over time by comparing the sentiment expressed in larger bodies of general comments/posts made at the beginning and at the end of the course.

Another potential limitation is that Warriner and colleagues’ [37] lemma database is based on rating words on an ordinal 1-to-9 scale, rather than a continuous interval scale. There may not be a uniform difference reflected between a rating of 1-to-2 as there is for 8-to-9. Very recently, new slide-rating methods for valence scores have been tested, which may provide a fruitful direction for future studies to utilise, if norms are developed [86].

The current study focussed on words used to describe feelings about death and dying, and has provided valuable insights into the way people feel about this issue. There is considerable potential for applying sentiment analysis to other related constructs, such as words chosen to describe feelings about Palliative Care, Advance Care Planning and Voluntary Assisted Dying. Such future research may help us better understand the nuances in community attitudes towards these important social policy issues. The possibilities of sentiment analysis as a methodology to detect emotional states of respondents also deserves future research attention. Previous studies have successfully used sentiment analysis to detect mental health conditions and level of threat in social media statements [17, 32]. Future research could pursue word sentiment as a method to detect emotional distress in health settings (e.g., carer bereavement risk), which could activate early intervention efforts.

## Conclusions

Using linguistic sentiment analysis of words chosen to describe death and dying was a valuable approach that extended our understanding of death attitudes and emotions in our sample. Sadness appears to permeate how we construe death, but analysis of the sentiment of words chosen to represent perceptions of others’ feelings towards death suggested that people perceive others to feel more negative about death than they do themselves. Furthermore, word sentiment analysis demonstrated that by the end of an online course about death, participants used more pleasant, calmer, and dominating words to express their feelings about death. This suggests that there was potentially a positive impact of the course on emotions and attitudes towards death, which requires future verification utilising a control group. The potential of applying sentiment analysis to related areas such as attitudes to Advance Care Planning and palliative care represent opportunities to develop a deeper understanding of emotions and attitudes on these topics of great interest to health policy.

## Supporting information

S1 Table Socio-demographic characteristics of MOOC enrolees, and socio-demographic characteristics and word sentiment scores of response sample at Time 1 (Baseline) and the sample retained at Time 2 (Complete cases at MOOC-end). (DOCX)

S2 Table Distribution of words that were matched in the wordlist of Warriner, Kuperman and Brybaert (2013), lemmatized, stemmed, hand corrected using a manually identified option and then matched in the wordlist, missing in the Warriner’s wordlist, or left blank by the participant. (DOCX)

S3 Table Comparison of word-choices made to describe personal feelings versus feelings of the Public, and word-choices made to describe personal feelings at baseline and MOOC-end. (DOCX)

S4 Table Bivariate relationships between socio-demographic variables and word sentiment scores at baseline and MOOC-end. (DOCX)

S1 File Sensitivity analyses using Multiple Imputation and complete-cases. (DOCX)

S1 Fig Distribution (normalised by number of words) of valence scores for all words from baseline self personally and the general public (Time 1, blue) and the total Warriner’s wordlist (orange). Baseline words in blue; Warriner’s wordlist in Orange. Words listed are 10 representative words selected from the maximum normalised count for each group.
(PDF)

S2 Fig Distribution (normalised by number of words) of arousal scores for all words from baseline self personally and the general public (Time 1, blue) and the total Warriner’s wordlist (orange). Baseline words in blue; Warriner’s wordlist in Orange. Words listed are 10 representative words selected from the maximum normalised count for each group.
(PDF)

S3 Fig Distribution (normalised by number of words) of dominance scores for all words from baseline self personally and the general public (Time 1, blue) and the total Warriner’s wordlist (orange). Baseline words in blue; Warriner’s wordlist in Orange. Words listed are 10 representative words selected from the maximum normalised count for each group.
(PDF)

S1 Dataset (XLSX)
