# Supplementary material for: Words describing feelings about death: A comparison of sentiment for self and others and changes over time
Source: PLoS One. 2021 Jan 6;16(1):e0242848. doi: 10.1371/journal.pone.0242848 (PMC7787376; doi:10.1371/journal.pone.0242848)
Supplement: S1 Table — (DOCX) [file pone.0242848.s001.docx]

**S1 Table. Socio-demographic Characteristics of MOOC Enrolees, and Socio-demographic Characteristics and Word Sentiment Scores of Response Sample at Time 1 (Baseline) and the Sample Retained at Time 2 (Complete cases at MOOC-end).**

|  | **All MOOC Enrolees (*n*=3116)** | | **Response Sample at Time 1 (*n*=1350)** | | | **Complete Cases Retained at Time 2 (*n*=582)** | | |
| --- | --- | --- | --- | --- | --- | --- | --- | --- |
|  | **Valid *n*** | ***%* or *M (SD), range*** | **Valid *n*** | ***%* or *M (SD), range*** | ***X^2^* or *t (p)^a^*** | **Valid *n*** | ***%* or *M (SD), range*** | ***X^2^* or *t (p)^b^*** |
| ***Socio-Demographic Characteristics at Enrolment*** |  |  |  |  |  |  |  |  |
| **Gender (female)** | 3105 | 92.4% | 1343 | 93.7% | 5.19 (.023) | 577 | 93.2% | 0.376 (.540) |
| **Age** | 3104 | 48.00 (12.38), 18-84 | 1347 | 49.50 (12.0), 18-84 | -5.96 (<.0005) | 580 | 50.07 (11.9) 19-81 | -1.54 (.123) |
| **Located in Australia** | 3116 | 81.3% | 1348 | 87.7% | 66.89 (<.0005) | 582 | 87.5% | 0.087 (.769) |
| **Self-identifies as a Health Professional** | 3112 | 71.3% | 1348 | 71.1% | .020 (.889) | 581 | 73.3% | 0.968 (.325) |
| **Has a University Qualification** | 3112 | 69.1% | 1348 | 68.5% | 0.289 (.591) | 581 | 66.6% | 1.626 (.202) |
| ***Word Sentiment scores*** |  |  |  |  |  |  |  |  |
| **Baseline Personal Words Valence score** | - | - | 1350 | 5.25 (1.31), 1.86-7.87 |  | 582 | 5.23 (1.32), 1.86-7.87 | .643 (.520) |
| **Baseline Others’ Words Valence score** | - | - | 1350 | 3.54 (0.91), 2.05-7.63 |  | 570 | 3.56 (0.95), 2.20-7.37 | .004 (.997) |
| **MOOC-End Personal Words Valence score** | - | - | - | - |  | 582 | 5.91 (1.10), 2.02-7.84 | - |
| **Baseline Personal Words Arousal score** | - | - | 1350 | 4.33 (0.58), 2.24-6.36 |  | 582 | 4.32 (0.58), 2.55-6.36 | .336 (.737) |
| **Baseline Others’ Words Arousal score** | - | - | 1350 | 4.82 (0.52), 3.20-6.15 |  | 570 | 4.83 (0.54), 3.20-6.11 | -.701 (.483) |
| **MOOC-End Personal Words Arousal score** | - | - | - | - |  | 582 | 4.18 (0.57), 2.55-5.69 | - |
| **Baseline Personal Words Dominance score** | - | - | 1350 | 5.14 (0.93), 3.05-7.14 |  | 582 | 5.15 (0.93), 3.05-7.14 | -.064 (.949) |
| **Baseline Others’ Words Dominance score** | - | - | 1350 | 4.11 (0.60), 2.86-7.07 |  | 570 | 4.13 (0.61), 2.91-6.76 | -.829 (.407) |
| **MOOC-End Personal Words Dominance score** | - | - | - | - |  | 582 | 5.65 (0.89), 2.66-7.20 | - |

*Notes.*

a. An examination of potential participation bias was conducted to identify socio-demographic differences between participants who commenced MOOC participation and completed the Time 1 activities (n=1350), and those lost to attrition between enrolment and Time 1 (n=1766). Overall, 1118 of these 1766 people never entered the MOOC after completing the enrolment form. There were statistically significant differences on age and location, with participants who completed the Time 1 activities being slightly older (*Cohen’s d* = .215) and more likely to be located in Australia (*Cohen’s d* = .296) than those lost to attrition.

b. An examination was conducted to identify differences between participants retained at Time 2 (*n*=582) and those lost to attrition between Time 1 and Time 2 (*n*=768) on socio-demographics and baseline word sentiment scores. No statistically significant differences were found (*p* >.05 for all analyses).
