# Supplementary material for: Words describing feelings about death: A comparison of sentiment for self and others and changes over time
Source: PLoS One. 2021 Jan 6;16(1):e0242848. doi: 10.1371/journal.pone.0242848 (PMC7787376; doi:10.1371/journal.pone.0242848)
Supplement: S2 Table — (DOCX) [file pone.0242848.s002.docx]

S2 Table. Distribution of words that were matched in the wordlist of Warriner, Kuperman and Brybaert (2013), lemmatized, stemmed, hand corrected using a manually identified option and then matched in the wordlist, missing in the Warriner’s wordlist, or left blank by the participant.

| **Linguistic Status of Words** | **Baseline Personal Words**  ***n*** | **Baseline Others’ Words**  ***n*** | **MOOC-End Personal Words**  ***n*** | **Total**  ***n*** |
| --- | --- | --- | --- | --- |
| **Original word used** | 3522 | 3659 | 1421 | 8602 |
| **Lemmatized original used** | 359 | 213 | 236 | 808 |
| **Stemmed original used** | 26 | 30 | 13 | 69 |
| **Manually identified lemma or stem used** | 301 | 171 | 161 | 633 |
| **Missing from Warriner’s and no alternative found** | 51 | 55 | 28 | 134 |
| **No original word provided (left blank)** | 73 | 174 | 31 | 278 |
| **Totals** | 4332 | 4302 | 1890 | 10524 |

*Note:* there are duplicate words in the counts (e.g. if two participants used the same word it would count as 2 for that word).
