# Supplementary material for: Words describing feelings about death: A comparison of sentiment for self and others and changes over time
Source: PLoS One. 2021 Jan 6;16(1):e0242848. doi: 10.1371/journal.pone.0242848 (PMC7787376; doi:10.1371/journal.pone.0242848)
Supplement: S3 Table — (DOCX) [file pone.0242848.s003.docx]

**S3 Table. Comparison of word-choices made to describe personal feelings versus feelings of the Public, and word-choices made to describe personal feelings at baseline and MOOC-end.**

| **Word exact-matches** | **Baseline Personal Words and Publics’ Words (*n*=1356)** | | **Baseline Personal Words and MOOC-end Personal Words (*n*=585)** | |
| --- | --- | --- | --- | --- |
|  | ***n*** | ***%*** | ***n*** | ***%*** |
| **None were the same** | 942 | 69.5% | 307 | 52.5% |
| **One word the same** | 293 | 21.6% | 185 | 31.6% |
| **Two words the same** | 72 | 5.3% | 66 | 11.3% |
| **All three words the same** | 49 | 3.6% | 27 | 4.6% |
