# Supplementary material for: Words describing feelings about death: A comparison of sentiment for self and others and changes over time
Source: PLoS One. 2021 Jan 6;16(1):e0242848. doi: 10.1371/journal.pone.0242848 (PMC7787376; doi:10.1371/journal.pone.0242848)
Supplement: S4 Table — (DOCX) [file pone.0242848.s004.docx]

**S4 Table. Bivariate Relationships between Socio-Demographic Variables and Word Sentiment Scores at Baseline and MOOC-end^a^.**

|  | **Socio-Demographic Characteristics** | | | | | | | |
| --- | --- | --- | --- | --- | --- | --- | --- | --- |
|  | **Australian Location** | | **Health Professional** | | **University Education** | | **Age** | |
|  | ***t(df)^b^*** | ***p*** | ***t(df)^b^*** | ***p*** | ***t(df)^b^*** | ***p*** | ***r ^b^*** | ***p*** |
| ***Word Sentiment Scores*** |  |  |  |  |  |  |  |  |
| **Baseline Personal Words Valence score** | 1.50 (2838.1) | .135 | 0.48 (9018.7) | .631 | 0.16 (6441.3) | .870 | .068 | .011* |
| **Baseline Personal Words Arousal score** | 2.59 (1029.3) | .010* | 3.11 (19378) | .002* | 0.38 (3125.3) | .703 | -.028 | .309 |
| **Baseline Personal Words Dominance score** | 2.38 (2101.2) | .018 | -.11 (7619.7) | .915 | 0.41 (10710) | .684 | .061 | .022 |
| **Baseline Others’ Words Valence score** | -0.29 (748.3) | .775 | -1.65 (1773.9) | .100 | 2.49 (1540.2) | .013* | .031 | .249 |
| **Baseline Others’ Words Arousal score** | 0.43 (902.1) | .671 | -0.39 (3702.5) | .697 | -.774 (2637.7) | .439 | -.003 | .923 |
| **Baseline Others’ Words Dominance score** | -1.07 (612.88) | .285 | -1.85 (1024.5) | .065 | 2.73 (887.3) | .006* | .039 | .158 |
| **MOOC-End Personal Words Valence score** | 1.61 (43.1) | .114 | -1.28 (39.27) | .208 | -0.42 (42.9) | .674 | -.077 | .048 |
| **MOOC-End Personal Words Arousal score** | 0.37 (70.7) | .715 | 0.28 (50.5) | .784 | -1.46 (43.76) | .153 | .241 | .485 |
| **MOOC-End Personal Words Dominance score** | 1.97 (38.59) | .057 | -1.46 (37.2) | .153 | 0.06 (41.3) | .950 | -.076 | .050 |

1. Analyses were based on imputed data, with a total n=1491 .
2. Dichotomous demographic variables were analysed using Independent samples t-test. Pooled results reported. Unequal variances were assumed due to unequal sample sizes in demographic variables (i.e., Welch’s t-test reported). Continuous variable were analysed using Pearson’s correlations. * *p* < .0166
