# Supplementary material for: Words describing feelings about death: A comparison of sentiment for self and others and changes over time
Source: PLoS One. 2021 Jan 6;16(1):e0242848. doi: 10.1371/journal.pone.0242848 (PMC7787376; doi:10.1371/journal.pone.0242848)
Supplement: S1 File — (DOCX) [file pone.0242848.s005.docx]

**S1 File. Sensitivity Analyses using Multiple Imputation.**

The prevalence of missing data in the dataset was examined, with sensitivity analyses undertaken. At Time 1 the number of participants that had supplied 3 words for Baseline Self was 1,368 out of the total 1,491 (8.25% missing), 1,413 supplying a valid word for the first entry, allowing for the calculation of 12,624 out of 13,419 (5.93% missing) individual valence, arousal, and dominance scores. For Baseline Others, 1,325 (11.13% missing) supplied words, whilst combining both Self and Others leads to only 1,261 complete cases providing 3 words for each activity, thus 15.43% of cases missing. Examining missing values at T2 reveals that there are only 591 participants that supplied valid 3 words for Self (60.36%) and examining across both T1 and T2 there are only 513 complete cases (65.59% missing). However, this only translates to 24.64% missing data for the individual valence, arousal, and dominance scores suggesting that participants completed some, but not all three words for each activity. The large number of cases missing from T2 is the ‘funnel of participation’ typical of MOOCs (Clow, 2013; Jordan, 2015; Padilla Rodriguez, 2020). The exact numbers also differ slightly from those reported in previous sections as computed scores used for analysis were averaged over the three words for each activity allowing for 1 missing word.

Multiple Imputation (MI) was conducted using fully conditional specification (FCS) Markov chain Monte Carlo (MCMC) method using SPSS to assess the impact of the missing values. The number of imputations was set to 20 and the maximum iteration to 1000 (Enders, 2010). The valance, arousal and dominance for each word of the activities from the Baseline Self, Baseline Others, and MOOC-end Self were set for imputation with all available demographic information (including age, gender, country, in Australia, health occupation, university qualification and responses to death questions) also included as independent, auxiliary variables. Primary research questions were tested using paired-samples t-tests with both complete-case and MI imputed data, given that SPSS provides statistical significance estimates for MI pooled data in paired-samples t-test (see Tables 1a and 2a for complete-case data, and Tables 1b and 2b for MI pooled data). The results indicated that means, standard errors, and effect sizes with MI data were similar to that utilising listwise deletion (complete-cases), which implied that overall the missing data was missing at random (not biased). The effect sizes were slightly smaller when using MI pooled data. A simulation study (Lee & Carlin, 2012) performed to examine the effectiveness of MI with various levels of missingness found multiple instances where MI was effective at 75% missingness, detecting slightly less bias when using MI pooled data compared to complete-cases. We therefore opted for reporting in the paper the results using MI pooled data. In line with recommendations (Lee & Carlin, 2012), we conducted complete-case data analysis in parallel to data analysis utilising the MI pooled function for all statistical analyses, and report complete-case results alongside the MI results in in this file (See Tables 3a, 3b, 4a and 4b). T-tests, correlations, and multiple regressions all obtained very similar results regardless of whether MI pooled data or complete-case data was used, and the overall conclusions were the same.

**Table 1a. Complete-Case Paired-Samples t-test Results: Word Sentiment Scores for self personally and for the general public, *n*=1350**

| **Outcome** | ***n*** | **Personal perspective**  ***Mean (SE); 95% CI*** | **Public perspective**  ***Mean (SE); 95% CI*** | ***Mean Diff. (SE);***  ***95% CI*** | **Perspective effects** | | |
| --- | --- | --- | --- | --- | --- | --- | --- |
|  |  |  |  |  | ***t (df)*** | ***p*** | ***Cohen’s d*** |
| Word Valence Score | 1350 | 5.25 (0.0356);  5.18 to 5.32 | 3.54 (0.024);  3.49 to 3.59 | 1.707 (0.0418) 1.6248 to 1.7891 | 40.77 (1349) | <.0005 | -1.112 |
| Word Arousal Score | 1350 | 4.33 (0.015);  4.30 to 4.36 | 4.82 (0.014);  4.79 to 4.85 | -0.494 (0.0208)  -0.5350 to -0.4532 | -23.69 (1349) | <.0005 | 0.645 |
| Word Dominance Score | 1350 | 5.14 (0.025);  5.09 to 5.19 | 4.11 (0.016);  4.08 to 4.14 | 1.030 (0.0293) 0.9726 to 1.0879 | 35.05 (1349) | <.0005 | -0.956 |

**Table 1b. Imputed Paired-Samples t-test Results: Word Sentiment Scores for self personally and for the general public, *n*=1491**

| **Outcome** | ***n*** | **Personal perspective**  ***Mean (SE); 95% CI*** | **Public perspective**  ***Mean (SE); 95% CI*** | ***Mean Diff. (SE);***  ***95% CI*** | **Perspective effects** | | |
| --- | --- | --- | --- | --- | --- | --- | --- |
|  |  |  |  |  | ***t (df)*** | ***p*** | ***Cohen’s d*** |
| Word Valence Score | 1491 | 5.25 (0.0351);  5.18 to 5.32 | 3.56 (0.026);  4.51 to 3.61 | 1.695 (0.0417);  1.61 to 1.77 | 40.67 (1882) | <.0005 | -1.048 |
| Word Arousal Score | 1491 | 4.33 (0.015);  4.31 to 4.36 | 4.82 (0.015);  4.79 to 4.85 | -0.484 (0.0210);  -0.5250 to -0.4430 | -23.03 (1085) | <.0005 | 0.600 |
| Word Dominance Score | 1491 | 5.16 (0.025);  5.10 to 5.19 | 4.12 (0.017);  4.06 to 4.15 | 1.026 (0.0287); 0.9700 to 1.0830 | 35.74 (4423) | <.0005 | -0.907 |

**Table 2a. Complete-Case Paired-Samples t-test Results: Personal Words Sentiment Scores at Baseline and MOOC-End, *n*=582**

| **Outcome** | ***n*** | **Baseline**  ***Mean (SE); 95% CI*** | **MOOC-End**  ***Mean (SE); 95% CI*** | ***Mean Diff. (SE);***  ***95% CI*** | **Time Effects** | | |
| --- | --- | --- | --- | --- | --- | --- | --- |
|  |  |  |  |  | ***t (df)*** | ***P*** | ***Cohen’s d*** |
| Word Valence Score | 582 | 5.23 (0.055);  5.12 to 5.33 | 5.91 (0.046);  5.82 to 6.00 | -0.687 (0.0571)  -0.7983 to -0.5739 | -12.01 (581) | <.0005 | 0.500 |
| Word Arousal Score | 582 | 4.32 (0.024);  4.27 to 4.37 | 4.18 (0.024);  4.13 to 4.22 | 0.148 (0.0304) 0.0879 to 0.2075 | 4.85 (581) | <.0005 | -0.201 |
| Word Dominance Score | 582 | 5.15 (0.039);  5.07 to 5.22 | 5.65 (0.037);  5.58 to 5.72 | -0.488 (0.0411)  -0.5699 to -0.4059 | -11.87 (581) | <.0005 | 0.469 |

**Table 2b. Imputed Paired-Samples t-test Results: Personal Words Sentiment Scores at Baseline and MOOC-End, *n*=1491**

| **Outcome** | ***n*** | ***Baseline***  ***Mean (SE); 95% CI*** | ***MOOC-End***  ***Mean (SE); 95% CI*** | ***Mean Diff. (SE);***  ***95% CI*** | **Time effects** | | |
| --- | --- | --- | --- | --- | --- | --- | --- |
|  |  |  |  |  | ***t (df)*** | ***P*** | ***Cohen’s d*** |
| Word Valence Score | 1491 | 5.25 (0.035);  5.183 to 5.321 | 5.90 (0.038);  5.82 to 5.98 | -0.652 (0.04541)  -0.7418 to -0.5622 | -14.36 (100) | <.0005 | 0.408 |
| Word Arousal Score | 1491 | 4.34 (0.015);  4.3 to 4.37 | 4.19 (0.023);  4.14 to 4.23 | 0.148 (0.02579) 0.0966 to 0.1991 | 5.74 (68) | <.0005 | -0.155 |
| Word Dominance Score | 1491 | 5.15 (0.025);  5.11 to 5.20 | 5.63 (0.037);  5.56 to 5.70 | -0.488 (0.04111)  -0.5670 to -0.4060 | -11.87 (53) | <.0005 | 0.339 |

**Table 3a. Complete-case Bivariate Relationships between Socio-Demographic Variables and Word Sentiment Scores at Baseline and MOOC-end^a^.**

|  | **Socio-Demographic Characteristics** | | | | | | | |
| --- | --- | --- | --- | --- | --- | --- | --- | --- |
|  | **Australian Location** | | **Health Professional** | | **University Education** | | **Age** | |
|  | ***t(df)^b^*** | ***p*** | ***t(df)^b^*** | ***p*** | ***t(df)^b^*** | ***p*** | ***r ^b^*** | ***p*** |
| ***Word Sentiment Scores*** |  |  |  |  |  |  |  |  |
| **Baseline Personal Words Valence score** | 1.76 (1346) | .078 | 0.59 (1346) | .553 | 0.14 (761.8) | .890 | .069 | .012* |
| **Baseline Personal Words Arousal score** | 2.59 (1346) | .010* | 2.83 (1346) | .005* | -0.10 (1346) | .919 | -.038 | .162 |
| **Baseline Personal Words Dominance score** | 2.36 (1346) | .019 | .064 (1346) | .949 | 0.50 (1346) | .616 | .058 | .034 |
| **Baseline Others’ Words Valence score** | -0.10 (1346) | .923 | -1.48 (831.7) | .140 | 2.01 (1346) | .045 | .021 | .432 |
| **Baseline Others’ Words Arousal score** | 0.49 (1346) | .625 | -0.20 (1346) | .841 | -.502 (1346) | .616 | -.005 | .842 |
| **Baseline Others’ Words Dominance score** | -1.00 (1346) | .316 | -1.95 (1346) | .051 | 2.44 (752.0) | .015* | .026 | .347 |
| **MOOC-End Personal Words Valence score** | 1.50 (580) | .135 | -1.13 (579) | .259 | -0.87 (347.4) | .386 | -.081 | .052 |
| **MOOC-End Personal Words Arousal score** | 0.65 (580) | .514 | 0.36 (579) | .718 | -1.39 (579) | .167 | .031 | .460 |
| **MOOC-End Personal Words Dominance score** | 1.62 (580) | .105 | -1.07 (579) | .287 | -0.22 (579) | .826 | -.093 | .025 |

1. Analysis of baseline sentiment variables were based on a sample size of 1350. Analysis of MOOC-end sentiment variables were based in a sample size of 582. A small number of participants were missing data on one of the demographic variables, therefore reducing the n in some analyses slightly.
2. Dichotomous demographic variables were analysed using Independent samples t-test. Where Levene’s test for equality of variances was significant, equal variances were not assumed. Continuous variable were analysed using Pearson’s correlations. * *p* < .0166

**Table 3b. Imputed Bivariate Relationships between Socio-Demographic Variables and Word Sentiment Scores at Baseline and MOOC-end^a^.**

|  | **Socio-Demographic Characteristics** | | | | | | | |
| --- | --- | --- | --- | --- | --- | --- | --- | --- |
|  | **Australian Location** | | **Health Professional** | | **University Education** | | **Age** | |
|  | ***t(df)^b^*** | ***p*** | ***t(df)^b^*** | ***p*** | ***t(df)^b^*** | ***p*** | ***r ^b^*** | ***p*** |
| ***Word Sentiment Scores*** |  |  |  |  |  |  |  |  |
| **Baseline Personal Words Valence score** | 1.50 (2838.1) | .135 | 0.48 (9018.7) | .631 | 0.16 (6441.3) | .870 | .068 | .011* |
| **Baseline Personal Words Arousal score** | 2.59 (1029.3) | .010* | 3.11 (19378) | .002* | 0.38 (3125.3) | .703 | -.028 | .309 |
| **Baseline Personal Words Dominance score** | 2.38 (2101.2) | .018 | -.11 (7619.7) | .915 | 0.41 (10710) | .684 | .061 | .022 |
| **Baseline Others’ Words Valence score** | -0.29 (748.3) | .775 | -1.65 (1773.9) | .100 | 2.49 (1540.2) | .013* | .031 | .249 |
| **Baseline Others’ Words Arousal score** | 0.43 (902.1) | .671 | -0.39 (3702.5) | .697 | -.774 (2637.7) | .439 | -.003 | .923 |
| **Baseline Others’ Words Dominance score** | -1.07 (612.88) | .285 | -1.85 (1024.5) | .065 | 2.73 (887.3) | .006* | .039 | .158 |
| **MOOC-End Personal Words Valence score** | 1.61 (43.1) | .114 | -1.28 (39.27) | .208 | -0.42 (42.9) | .674 | -.077 | .048 |
| **MOOC-End Personal Words Arousal score** | 0.37 (70.7) | .715 | 0.28 (50.5) | .784 | -1.46 (43.76) | .153 | .241 | .485 |
| **MOOC-End Personal Words Dominance score** | 1.97 (38.59) | .057 | -1.46 (37.2) | .153 | 0.06 (41.3) | .950 | -.076 | .050 |

1. Analyses were based on imputed data, with a total n=1491 .
2. Dichotomous demographic variables were analysed using Independent samples t-test. Pooled results reported. Unequal variances were assumed due to unequal sample sizes in demographic variables (i.e., Welch’s t-test reported). Continuous variable were analysed using Pearson’s correlations. * *p* < .0166

Table 4a. Complete-Case Hierarchical Multiple Linear Regressions Predicting MOOC-End Sentiment scores, adjusting for Baseline sentiment scores (*n*=580)^a^

|  | **MOOC-End Sentiment Scores** | | | | | | | | |
| --- | --- | --- | --- | --- | --- | --- | --- | --- | --- |
|  | **Word Valence Score** | | | **Word Arousal Score** | | | **Word Dominance Score** | | |
| Baseline Predictor Variables | ***Unstandardised B (Standardised β)*** | ***B 95%CI*** | ***p*** | ***Unstandardised B (Standardised β)*** | ***B 95%CI*** | ***p*** | ***Unstandardised B (Standardised β)*** | ***B 95%CI*** | ***p*** |
| ***Initial Model Variables:*** |  |  |  |  |  |  |  |  |  |
| **Baseline Sentiment score** | .301 (**.360**) | .237 to .365 | <.0005 | .186 (**.191**) | .108 to .265 | <.0005 | .288 (**.300**) | .213 to .362 | <.0005 |
| ***Final Model Variables:*** |  |  |  |  |  |  |  |  |  |
| **Baseline Sentiment score** | .308 (**.368**) | .245 to .372 | <.0005 | .188 (**.193**) | .109 to .267 | <.0005 | .295 (**.308**) | .221 to .370 | <.0005 |
| **Age** | -.010 (**-.104**) | -.017 to -.002 | .010 | .002 (.051) | -.002 to .006 | .231 | -.008 (**-.112**) | -.014 to -.002 | .006 |
| **Located in Australia (Yes)** | -.180 (-.054) | -.435 to .075 | .166 | -.018 (-.011) | -.158 to .122 | .796 | -.154 (-.057) | -.365 to .057 | .151 |
| **Health Professional (Yes)** | .106 (.042) | -.089 to .300 | .287 | .015 (.012) | -.092 to .123 | .779 | .079 (.039) | -.082 to .240 | .336 |
| **University Education (Yes)** | .025 (.012) | -.155 to .204 | .788 | .074 (.061) | -.024 to .173 | .139 | -.023 (-.012) | -.170 to .125 | .765 |
| ***Model Fit Statistics:*** |  |  |  |  |  |  |  |  |  |
| ***Initial R^2^*** | .130 |  |  | .036 |  |  | .090 |  |  |
| ***F*** | 86.10 (*p*<.0005) |  |  | 21.88 (*p*<.0005) |  |  | 57.27 (*p*<.0005) |  |  |
| ***ΔR^2^*** | .017 |  |  | .006 |  |  | .018 |  |  |
| ***ΔF*** | 2.88 (*p*=.022) |  |  | 0.83 (*p*=.505) |  |  | 2.93 (*p*=.020) |  |  |
| ***Final Model p*** | <.0005 |  |  | <.0005 |  |  | <.0005 |  |  |

*Notes.*

^a^ Of the 582 complete cases with data on sentiment at both assessments, 2 cases were missing data on at least one of the socio-demographic variables. Therefore the multiple regression had a total n of 580.

Table 4b. Imputed Hierarchical Multiple Linear Regressions Predicting MOOC-End Sentiment scores, adjusting for Baseline sentiment scores (*n*=1491)^a^

|  | **MOOC-End Sentiment Scores** | | | | | | | | |
| --- | --- | --- | --- | --- | --- | --- | --- | --- | --- |
|  | **Word Valence Score** | | | **Word Arousal Score** | | | **Word Dominance Score** | | |
| Baseline Predictor Variables | ***Unstandardised B (SE)*** | ***B 95%CI*** | ***p*** | ***Unstandardised B (SE)*** | ***B 95%CI*** | ***p*** | ***Unstandardised B (SE)*** | ***B 95%CI*** | ***p*** |
| ***Initial Model Variables:*** |  |  |  |  |  |  |  |  |  |
| **Baseline Sentiment score** | .313 (.036) | .241 to .385 | <.0005 | .193 (.046) | .099 to .287 | <.0005 | .302 (.042) | .218 to .386 | <.0005 |
| ***Final Model Variables:*** |  |  |  |  |  |  |  |  |  |
| **Baseline Sentiment score** | .317 (.035) | .246 to .389 | <.0005 | .196 (.047) | .101 to .291 | <.0005 | .302 (.041) | .220 to .384 | <.0005 |
| **Age** | -.008 (.003) | -.015 to -.002 | .015 | .002 (.002) | -.002 to .005 | .319 | -.006 (.003) | -.012 to -.001 | .024 |
| **Located in Australia (Yes)** | -.171 (.138) | -.449 to .107 | .233 | .005 (.059) | -.113 to .123 | .929 | -.184 (.117) | -.420 to .052 | .124 |
| **Health Professional (Yes)** | .114 (.106) | -.100 to .329 | .290 | .011 (.051) | -.091 to .113 | .832 | .109 (.088) | -.069 to .286 | .224 |
| **University Education (Yes)** | .017 (.098) | -.181 to .214 | .865 | .080 (.050) | -.021 to .180 | .118 | -.025 (.081) | -.187 to .138 | .761 |
| ***Model Fit Statistics (Ranges in 20 imputed datasets) ^a^:*** |  |  |  |  |  |  |  |  |  |
| ***Initial R^2^ range*** | .094 to .201 |  |  | .013 to .068 |  |  | .058 to .125 |  |  |
| ***F range*** | 153.23 to 373.22 (all *p*<.0005) |  |  | 19.47 to 108.71 (all *p*<.0005) |  |  | 91.11 to 211.27 (all *p*<.0005) |  |  |
| ***ΔR^2^ range*** | .005 to .028 |  |  | .001 to .019 |  |  | .003 to .030 |  |  |
| ***ΔF range*** | 2.17 to 12.26 (*p*=.000 to 007) |  |  | 0.50 to 7.31 (*p*=.000 to .733) |  |  | 1.20 to 12.92 (*p*=.000 to .311) |  |  |
| ***Final Model p range*** | All <.0005 |  |  | All <.0005 |  |  | All <.0005 |  |  |

*Notes.*

^a^  Pooled estimates of Model fit are not provided in SPSS. So we have reported the range of statistics obtained in the 20 imputed datasets.
