# Supplementary material for: Words describing feelings about death: A comparison of sentiment for self and others and changes over time
Source: PLoS One. 2021 Jan 6;16(1):e0242848. doi: 10.1371/journal.pone.0242848 (PMC7787376; doi:10.1371/journal.pone.0242848)
Supplement: S3 Fig — Baseline words in blue; Warriner’s wordlist in Orange. Words listed are 10 representative words selected from the maximum normalised count for each group. (PDF) [file pone.0242848.s008.pdf]

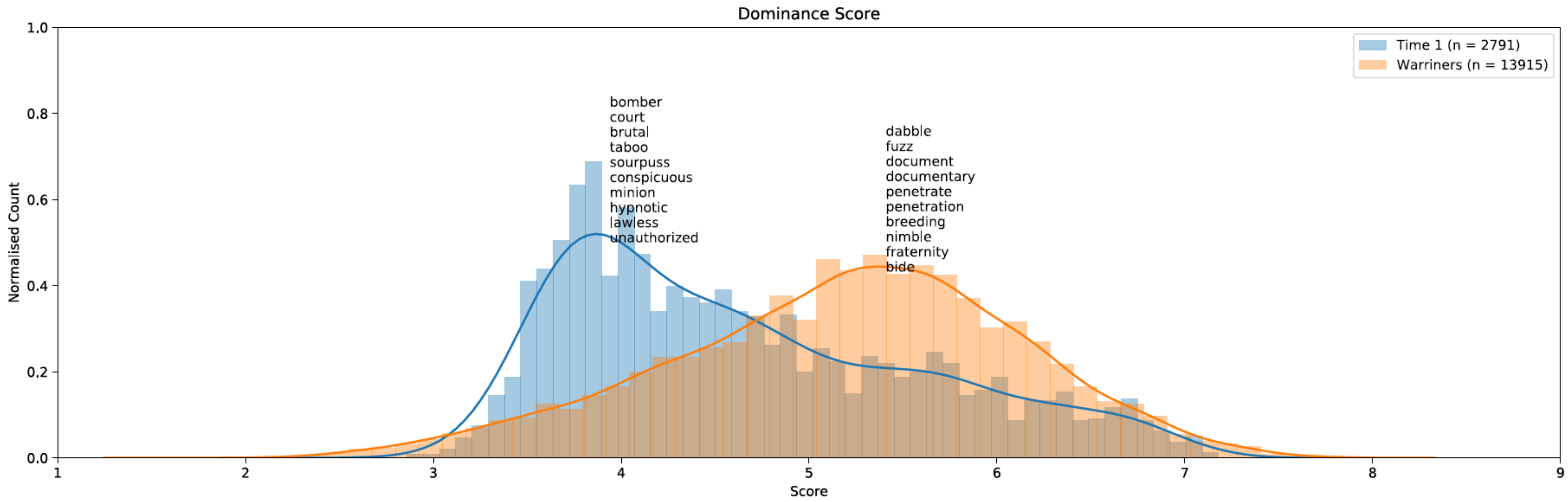

S1 Fig. Distribution (normalised by number of words) of Dominance score for all words from baseline self personally and the general public (Time 1, blue) and the total Warriner's wordlist (orange). Words listed are 10 representative words selected from the maximum normalised count for each group.
